# Haemoptysis from a pulmonary arteriovenous malformation in a post molar pregnancy gestational trophoblast tumour patient managed by radiological embolisation: a case report

**DOI:** 10.1186/1752-1947-8-117

**Published:** 2014-04-03

**Authors:** Zoë McDonald-Burrows, Rhian Davies, Elizabeth Goode, Candice Clarke, James Jackson, Michael Seckl, Philip Savage

**Affiliations:** 1 Ealing Hospital Trust, Southall UB1 3HW, UK; 2 Velindre Cancer Centre, Cardiff CF14 2TL, UK; 3 Gastroenterology Dept, Norfolk and Norwich University Hospital, Norwich NR4 7UY, UK; 4 Renal Unit, Hammersmith Hospital, London W12 0HS, UK; 5 Department of Imaging, Hammersmith Hospital, London W12 0HS, UK; 6 Charing Cross Hospital Department for Oncology, Hammersmith, London W6 8RF, UK; 7 BCCA Vancouver Island, Victoria, BC V8R 6V5, Canada

**Keywords:** AVM, Embolisation, Haemoptysis, hCG, Trophoblast

## Abstract

**Introduction:**

Gestational trophoblastic tumours are a rare form of malignancy, which in the majority of cases arise from abnormal trophoblast cells formed in a complete molar pregnancy. These tumours are extremely sensitive to chemotherapy and high cure rates approaching 100% can be expected. The disease is usually limited to the uterus where the abnormal trophoblast proliferation and human chorionic production can lead to vascular changes including the formation of arteriovenous malformations.

**Case presentation:**

We describe the case of a 28-year-old Caucasian woman who presented to the United Kingdom's Gestational Trophoblast Tumour Service with rising human chorionic gonadotropin levels following a uterine evacuation for a complete molar pregnancy. She was commenced on chemotherapy but subsequently reported two episodes of haemoptysis. Computed tomography imaging demonstrated findings consistent with a pulmonary arteriovenous malformation, probably due to a small pulmonary metastasis, complicated by recent haemorrhage. These findings were confirmed on emergency pulmonary arteriography, and the pulmonary arteriovenous malformation was successfully embolised.

**Conclusions:**

Arteriovenous malformations secondary to gestational trophoblastic tumours at metastatic sites have only been reported in a very limited number of cases. When significant bleeding occurs, as in this case of a pulmonary lesion, urgent referral for embolisation is indicated.

## Introduction

Gestational trophoblastic tumours (GTT) are characteristically highly vascular and can produce heavy or even life-threatening bleeding from the uterus or sites of metastatic disease [1]. Over 90% of cases of GTT result from the continual growth of the abnormal trophoblast cells that are formed in a complete molar pregnancy. Molar pregnancies are rare and occur at a rate of approximately one for every 600 conceptions and carry an approximate risk of malignant change of 14% for complete moles and 1% for partial moles [2].

The genetically abnormal malignant trophoblast cells share many of the characteristics of healthy trophoblast cells, including human chorionic gonadotropin (hCG) production, rapid division, the ability to invade surrounding tissues and to stimulate angiogenesis and other vascular changes. Fortunately gestational trophoblast tumours are extremely sensitive to chemotherapy and modern cure rates approaching 100% can be expected for patients who develop GTT after a molar pregnancy [3].

In the majority of GTT cases the disease is limited to the uterus where the abnormal trophoblast proliferation and localised hCG production may lead to focal vascular changes including the formation of arteriovenous malformations (AVMs) [4,5]. The development of uterine AVMs is well characterised in patients with GTTs and while many are asymptomatic, some can lead to life-threatening bleeding requiring treatment with hysterectomy or radiological embolisation [1].

The most common site of metastasis in GTT are the lungs, but the formation of AVMs at this or any other extra-uterine site of spread has only been recorded rarely [6,7]. This case reviews the diagnosis of a pulmonary AVM (pAVM) in a patient with GTT and occult lung metastases successfully treated with radiological embolisation and chemotherapy.

## Case presentation

A 28-year-old Caucasian woman underwent a uterine evacuation for a molar pregnancy at her local hospital and was registered for follow up with the United Kingdom (UK) Gestational Trophoblast Tumour service. The histopathology review confirmed the diagnosis of a complete molar pregnancy and as such she was enrolled in the hCG surveillance programme. After an initial early fall in her serum hCG level, her hCG level rose in two consecutive samples, because of this she was reviewed in clinic 14 weeks post-evacuation prior to consideration of chemotherapy treatment.

**Figure 1:**
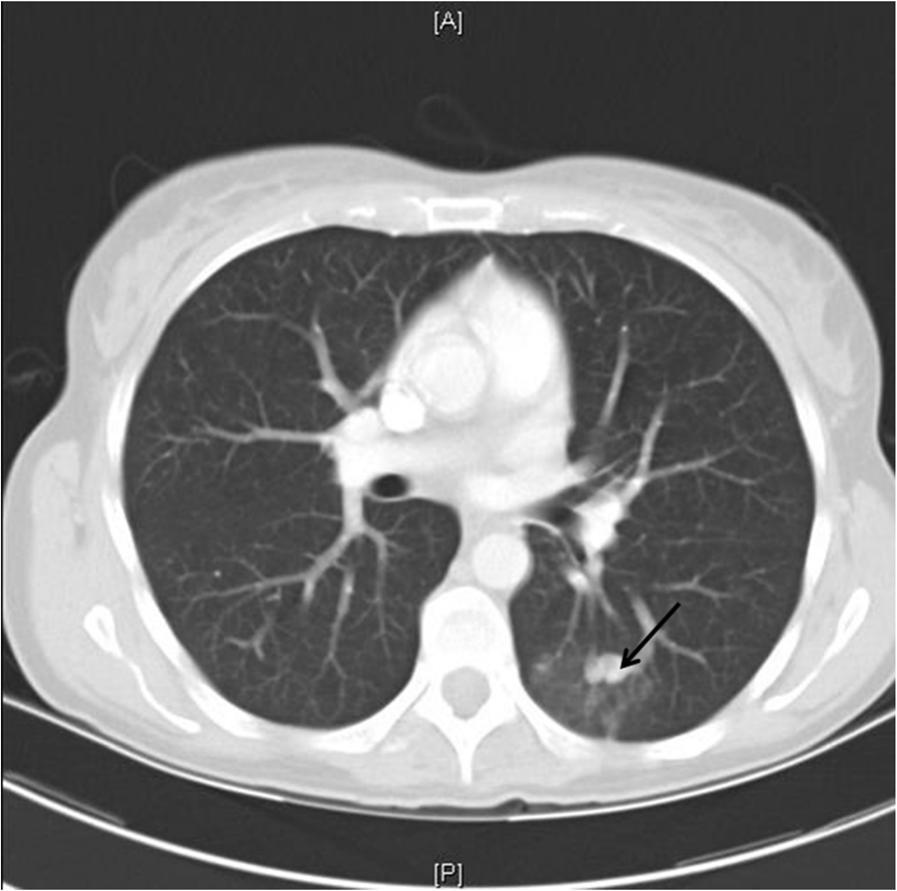
Axial computed tomography image at level of aortic root demonstrates small left lower lobe pulmonary arteriovenous malformation (arrow) with surrounding ground glass opacity consistent with recent haemorrhage.

**Figure 2:**
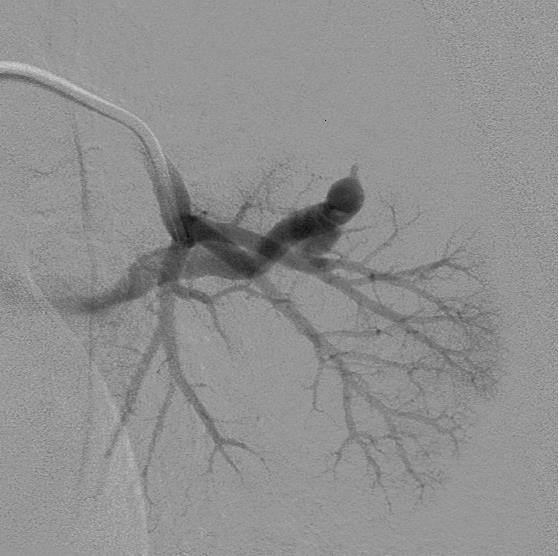
Selective left pulmonary artery branch angiogram demonstrates rapid arteriovenous shunting through the pulmonary arteriovenous malformation.

**Figure 3:**
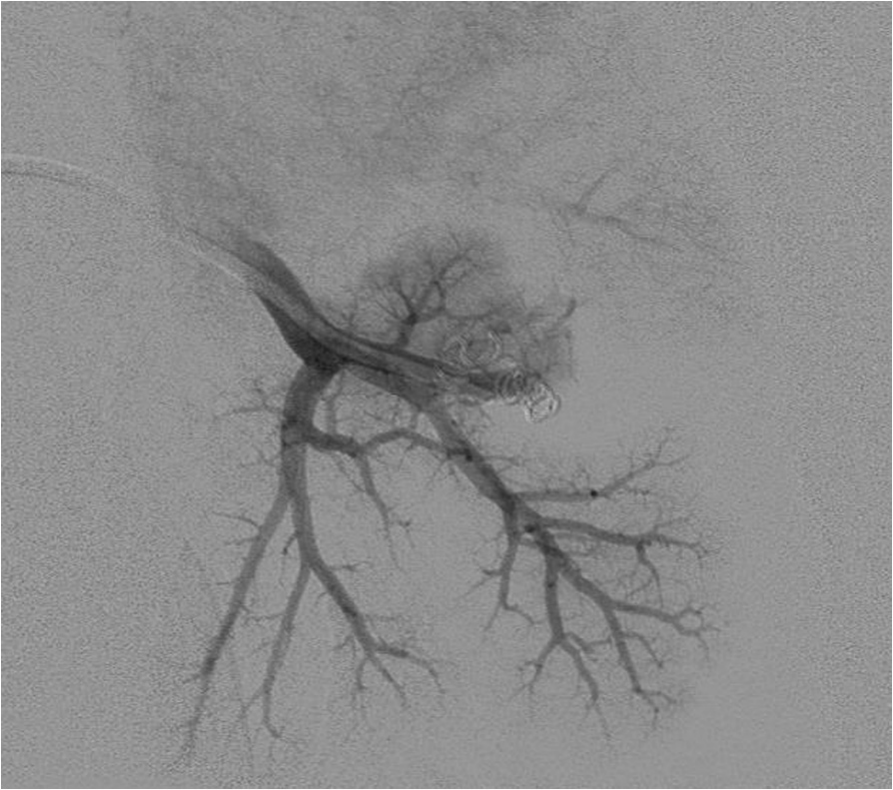
Postembolization pulmonary arteriogram demonstrates complete occlusion of the pulmonary arteriovenous malformation.

**Figure 4:**
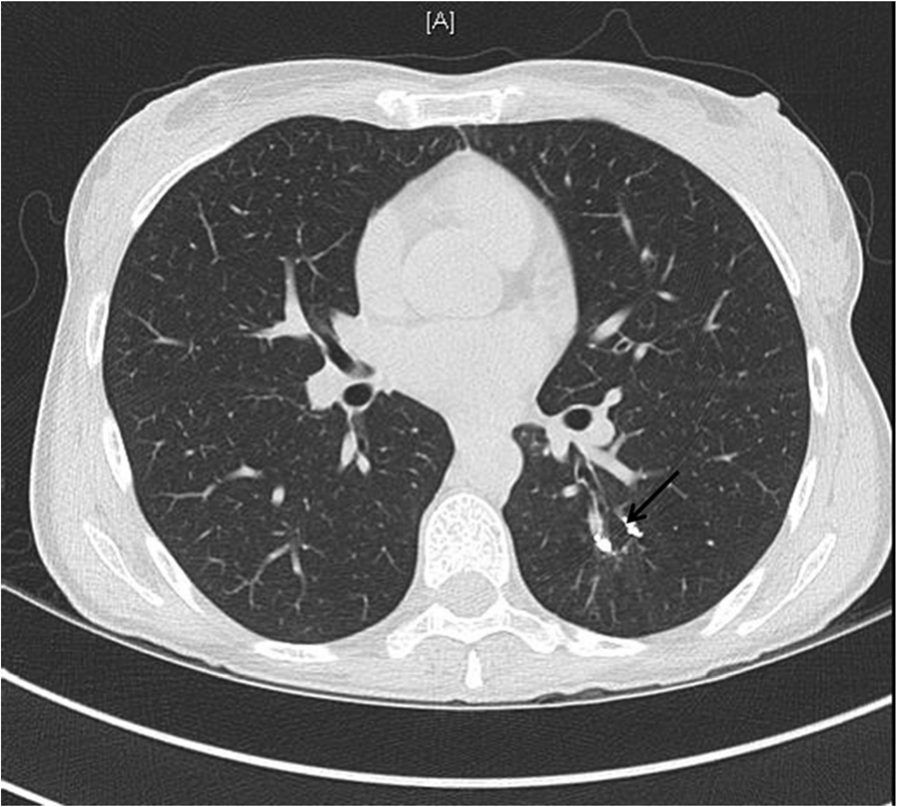
**Axial computed tomography image at same level as Figure**1**performed 4 years after embolization shows metallic coils and obliteration of pulmonary arteriovenous malformation (arrow).**

She had an unremarkable medical history, with two normal pregnancies, no major surgery or illnesses and no regular medications. The routine investigations demonstrated an hCG value of 2070IU/L, a normal chest X-ray and no visible uterine mass on the pelvic Doppler ultrasound but some increased vascularity. These results confirmed the indications for treatment and produced an International Federation of Gynecology and Obstetrics (FIGO) prognostic score of 1. Following the UK’s standard treatment protocols, chemotherapy treatment was commenced with the low-risk regimen of methotrexate and folinic acid [3].The patient was well 6 weeks after the commencement of chemotherapy but reported two modest episodes of haemoptysis and was readmitted for emergency investigation. Initial blood tests confirmed a normal clotting screen and platelet count, whereas a computed tomography (CT) scan of her thorax demonstrated a 17mm lobulated nodule in the apical segment of her left lower lobe. Surrounding this lesion, there was patchy ground glass opacification consistent with pulmonary haemorrhage (Figure 1). The CT findings were typical of a pAVM, probably due to a small pulmonary metastasis, complicated by recent haemorrhage.An urgent referral was made to the interventional radiology team and emergency pulmonary arteriography confirmed the presence of a pAVM (Figure 2) with two separate feeding vessels. The pAVM was successfully embolised with magnetic resonance-compatible coils as shown in Figure 3 and led to resolution of the haemoptysis. A follow-up thoracic CT 4 years after embolisation confirmed that the pAVM had been cured as shown in Figure 4.

**Figure 5:**
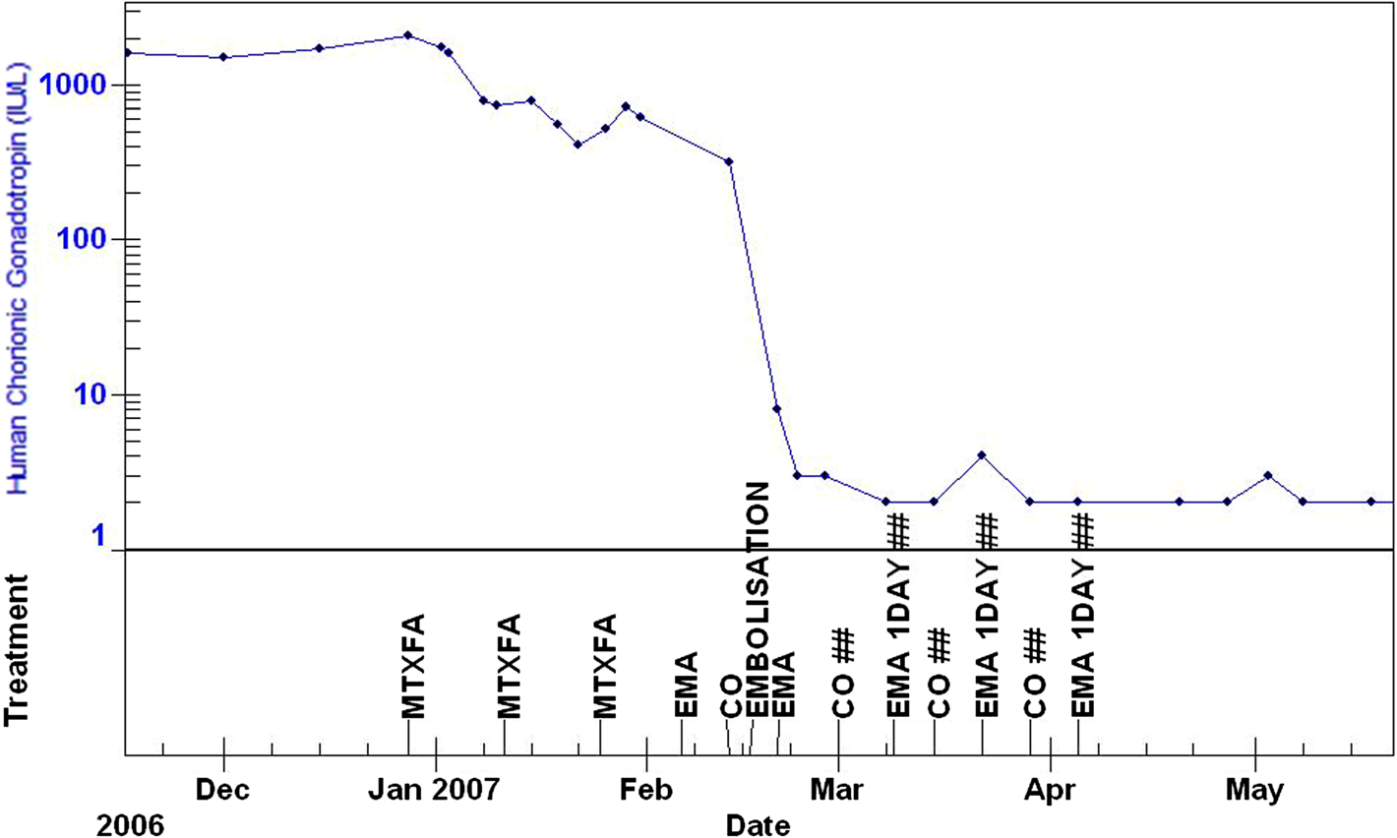
**Patient human chorionic gonadotropin levels during course of treatment.** Levels fell at a slow rate with methotrexate and treatment was changed to the etoposide, methotrexate and dactinomycin alternating with cyclophosphamide and vincristine regime. The human chorionic gonadotropin levels normalised 3 weeks subsequent to this change. Abbreviations: CO, cyclophosphamide and vincristine; EMA, etoposide, methotrexate and dactinomycin; HCG(S), human chorionic gonadotropin.

Just prior to the embolisation procedure, her chemotherapy treatment had been changed to the etoposide, methotrexate and dactinomycin alternating with cyclophosphamide and vincristine regime as a result of a slow rate of hCG fall with methotrexate. Subsequent to this change, her hCG levels normalised 3 weeks later and after an additional 6 weeks of treatment chemotherapy was completed as shown in Figure 5. She remains well 4 years later, is cured of her tumour, has gone on to have another healthy baby and has had no further episodes of haemoptysis.

## Discussion

Gestational trophoblast tumours are a rare but highly curable form of malignancy. The majority of cases occur as a result of a complete molar pregnancy and are characterised as hCG producing, highly vascular and chemosensitive tumours. The effect of the tumour-derived hCG on angiogenesis is well documented [4,5] and AVMs in the uterus are a relatively frequent event and generally simple to manage with uterine artery embolisation for cases with heavy or persistent bleeding [1].

GTT metastases at other anatomical sites may also be highly vascular and have the potential to form AVMs that can lead to life-threatening haemorrhage [6,7]. In this current case a patient with low-risk post-molar pregnancy GTT developed haemoptysis during methotrexate chemotherapy and imaging investigations demonstrated an AVM in her left lung. The most probable cause was that this was as a result of a small GTT metastasis that was too small to be visualised on the initial screening chest X-ray. The presentation, timing and clinical findings were consistent with this being a GTT-related AVM rather than other aetiology. The vast majority of pAVMs occur in patients with hereditary haemorrhagic telangiectasia in whom they are usually multiple. In this patient there was no family history or clinical evidence of this condition, in addition, no other pAVMs were present in either lung.

In uterine AVMs with light to moderate bleeding, the usual management is to manage these expectantly as many resolve spontaneously with successful chemotherapy treatment. However, in this case with a pulmonary lesion and the potential for life-threatening bleeding, we elected to control the bleeding risk promptly by performing the embolisation.

In the UK the management of patients with post-molar pregnancy GTT is centralised, which has allowed the development of expertise in these rare cases. From the almost 3000 other patients treated previously, no cases of bleeding from pAVMs have been recorded. As a result the FIGO recommendation of a chest X-ray remains the appropriate investigation for assessment of potential lung metastases in these patients [8]. Clearly if haemoptysis occurs, as in this rare case, then more detailed assessment with CT is indicated.

## Conclusions

Although we appreciate that pAVMs secondary to metastatic GTT are extremely rare, it is clear from this report that they are at risk of rupture causing haemoptysis. When diagnosed, we suggest that urgent referral for embolisation should be considered to reduce the risk of bleeding.

## Consent

Written informed consent was obtained from the patient for publication of this case report and accompanying images. A copy of the written consent is available for review by the Editor-in-Chief of this journal.

## Abbreviations

AVM: arteriovenous malformation; CT: computed tomography; FIGO: International Federation of Gynecology and Obstetrics; GTT: gestational trophoblastic tumour; hCG: human chorionic gonadotropin; pAVM: pulmonary arteriovenous malformation; MTXFA: Methotrexate and Folnic Acid; EMA: Etoposide, Methotrexate and Dactinomycin; CO: Cyclophosphamide and Vincristine.

## Competing interests

The authors declare that they have no competing interests.

## Authors’ contributions

All the authors have read and approved the final version of this manuscript. RD, EG, CC and PS were involved in writing the manuscript. RD and ZMB compiled the final draft of the manuscript. PS revised the manuscript critically and was a major contributor in writing the manuscript. JJ provided the radiological input for the case and prepared the images. EG, CC, JJ, MS and PS were involved in the clinical care of the patient. PS is the corresponding author.
